# Preparation and Physical Properties of Quaternary Mn_2_FeSi_0.5_Al_0.5_ Alloy Powders with Heusler and β-Mn Structures

**DOI:** 10.3390/ma18020309

**Published:** 2025-01-11

**Authors:** Katerina Skotnicova, Jan Jurica, Ondrej Zivotsky, Tomas Cegan, Kamila Hrabovska, Vlastimil Matejka, Simona Zla, Monika Kawulokova, Artur Chrobak

**Affiliations:** 1 Faculty of Materials Science and Technology, VSB—Technical University of Ostrava, 17. listopadu 2172/15, 70 800 Ostrava, Czech Republic; katerina.skotnicova@vsb.cz (K.S.); jan.jurica@vsb.cz (J.J.); vlastimil.matejka@vsb.cz (V.M.); simona.zla@vsb.cz (S.Z.); monika.kawulokova@vsb.cz (M.K.); 2 Faculty of Electrical Engineering and Computer Science, VSB—Technical University of Ostrava, 17. listopadu 2172/15, 708 00 Ostrava, Czech Republic; ondrej.zivotsky@vsb.cz (O.Z.); kamila.hrabovska@vsb.cz (K.H.); 3 Institute of Materials Engineering, University of Silesia in Katowice, 75 Pułku Piechoty 1A, 41-500 Chorzów, Poland; artur.chrob@gmail.com

**Keywords:** Mn-based alloys, powders, ball milling, microstructure, differential thermal analysis, antiferromagnetism

## Abstract

Manganese-based alloys with the composition Mn_2_FeZ (Z = Si, Al) have been extensively investigated in recent years due to their potential applications in spintronics. The Mn_2_FeSi alloy, prepared in the form of ingots, powders, or ribbons, exhibits either a cubic full-Heusler (*L*2_1_) structure, an inverse-Heusler (XA) structure, or a combination of both. In contrast, the Mn_2_FeAl alloy has so far been synthesized only in the form of ingots, featuring a primitive cubic (β-Mn type) structure. This study focuses on the new quaternary Mn_2_FeSi_0.5_Al_0.5_ alloy synthesized from pure Mn, Fe, Si, and Al powders via mechanical alloying. The elemental powders were ball-milled for 168 h with a ball-to-powder ratio of 10:1, followed by annealing at 550 °C, 700 °C, and 950 °C for 8 h in an argon protective atmosphere. The results demonstrate that annealing at lower temperatures (550 °C) led to the formation of a Heusler structure with a lattice constant of 0.5739 nm. Annealing at 700 °C resulted in the coexistence of several phases, including the Heusler phase and a newly developed primitive cubic β-Mn structure. Further increasing the annealing temperature to 950 °C completely suppressed the Heusler phase, with the β-Mn structure, having a lattice constant of 0.6281 nm, becoming the dominant phase. These findings confirm the possibility of tuning the structure of Mn_2_FeSi_0.5_Al_0.5_ alloy powder—and thereby its physical properties—by varying the annealing temperature. The sensitivity of magnetic properties to structural changes is demonstrated through magnetization curves and zero-field-cooled/field-cooled curves in the temperature range of 5 K to 300 K.

## 1. Introduction

Heusler alloys are currently the focus of intensive research due to their intriguing physical properties, which enable applications across various scientific fields, including spintronics [1,2], sensorics [3], microelectronics [4], magnetic refrigeration [2,5], thermoelectric systems, and other energy conversion technologies [6]. A particularly noteworthy class consists of manganese-based Heusler alloys, primarily recognized for their antiferromagnetic behaviour. The internal spin compensation in these systems leads to a relatively small total magnetic moment, which minimizes stray fields and makes devices based on these materials less susceptible to external magnetic interference. These properties render Mn-based Heusler alloys ideal as spin injectors for magnetic random-access memories and other spin-dependent devices [7]. Additionally, Mn-based Heusler alloys have garnered significant attention not only for their antiferromagnetic properties but also for their potential applications in spin-transfer torque, the spin Hall effect, non-collinear magnetism, and rare-earth-free hard magnets [8,9].

The physical properties of Heusler alloys often vary significantly depending on the preparation method employed. A considerable portion of studies focuses on bulk alloys produced using conventional arc or induction melting techniques, or on thin films sputtered onto various substrates [10,11]. Studies involving (nano)crystalline ribbons [12] or glass-coated microwires [13] are relatively uncommon. Powder samples are occasionally investigated, typically obtained either by crushing ingots prepared via arc melting followed by grinding in a ball mill [14,15], or by mechanical alloying of pure elemental powders [16,17,18]. In the case of Mn-based Heusler alloys, theoretical studies dominate [19]. When these systems are experimentally synthesized, they are most often prepared in the form of bulk ingots.

In this work, we build on our previous study [20], where Mn_2_FeSi powder alloy was synthesized from pure elemental powders via mechanical alloying (168 h of milling) and subsequently annealed at 1173 K for 1.5 h to form an inverse-Heusler (XA) structure. These findings were further supported by additional studies focusing on Mn_2_FeSi ingots prepared through arc/induction melting [21,22] and on rapidly quenched ribbons [23]. However, in arc-melted Mn_2_FeSi ingots, a minor cubic β-Mn structure was observed alongside the dominant cubic Heusler structure [19]. It is known that Mn_2_FeAl ingots crystallize in a geometrically frustrated cubic β-Mn structure and exhibit antiferromagnetic behaviour at low temperatures [22,24]. This provides motivation to explore the synthesis of a quaternary Mn_2_FeSi_0.5_Al_0.5_ alloy to study the competition between the Heusler and β-Mn structures during the preparation process and subsequent thermal homogenization. Notably, this quaternary alloy has been cast in the form of an ingot, and its structural and magnetic properties appear to be similar to those of Mn_2_FeAl [25]. However, the different preparation technology and thermal conditions can markedly influence the origin of the Heusler and/or β-Mn structure. In this paper, we report on the synthesis of the new Mn_2_FeSi_0.5_Al_0.5_ powder alloy from raw materials, investigating its structural transformations as a function of annealing temperature. Furthermore, we document the fundamental differences in structural and magnetic properties between powders and ingots, highlighting the impact of different preparation techniques.

## 2. Materials and Methods

### 2.1. Starting Materials

**Figure 1:**
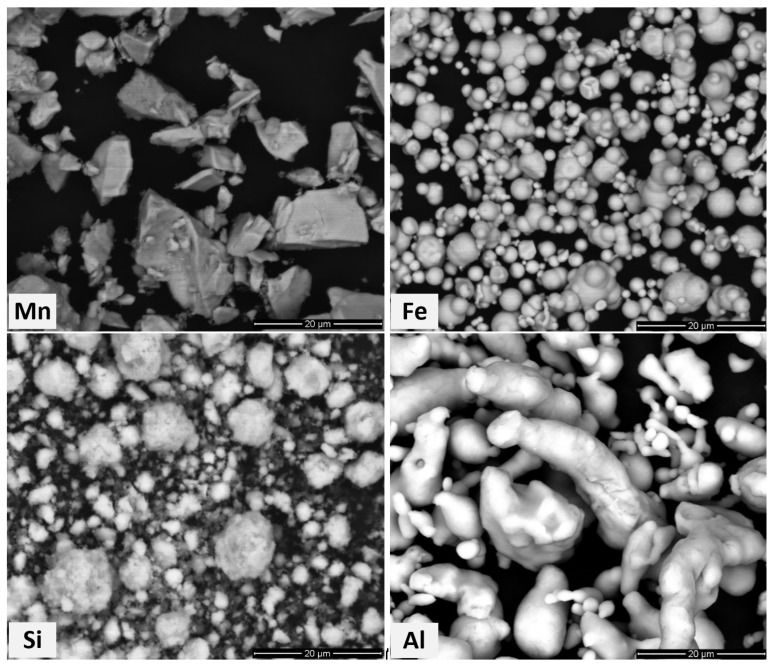
Back-scattered electron (BSE) micrographs of starting powders.

The powders of manganese (≥99.3%, D50 = 29.8 µm), iron (≥99.5%, D50 = 6.4 µm), silicon (99.9985%, D50 = 5.5 µm), and aluminium (99.5%, D50 = 11.2 µm) from Thermo Fisher Scientific (Waltham, MA, USA) were used as starting materials. The Mn:Fe:Si:Al ratio corresponds to 50:25:12.5:12.5 (in at.%). The particle morphologies of Mn, Fe, Si, and Al powders are shown in Figure 1. All powder particles exhibited irregular shapes with varying surface roughness, determined by their respective production methods.

### 2.2. Synthesis

The Mn_2_FeSi_0.5_Al_0.5_ alloy powder was prepared through mechanical alloying followed by thermal treatment. Mn, Fe, Si, and Al powders were initially mixed using a Turbula T2F mixer (WILLY A. BACHOFEN AG, Muttenz, Switzerland), rotated at 50 RPM for 15 min, and placed in a stainless-steel milling bowl along with 5 mm stainless steel balls (ball-to-powder weight ratio of 10:1). The n-Hexane was added as a process control agent (PCA) to prevent cold welding and bonding between powder particles and to ensure good homogeneity. The milling bowl was sealed, evacuated several times, and subsequently filled with argon gas (99.999%). Mechanical alloying was conducted at 500 RPM for 168 h using a high-energy ball mill E-max (Retsch GmbH, Haan, Germany), which features an integrated water-cooling system for the milling bowl. Based on the results of XRD and differential thermal analysis (DTA), annealing of the as-prepared powder was performed at various temperatures using a sintering furnace (Xerion Advanced Heating, Ofentechnik, Germany). The study details the structural evolution of the Mn_2_FeSi_0.5_Al_0.5_ alloy powder during annealing at 550 °C (823 K), 700 °C (973 K), and 950 °C (1223 K) for 8 h in an argon protective atmosphere, with a heating rate of 10 °C/min followed by furnace cooling.

### 2.3. Structural, Chemical, and Phase Analysis

The morphology and microstructure of the samples were investigated using a Quanta 450 FEG scanning electron microscope (SEM, FEI Company, Fremont, CA, USA) equipped with an energy-dispersive spectroscopy (EDS, EDAX Inc., Mahwah, NJ, USA). The crystal structure and phase composition of the powders were investigated at room temperature (RT) using a Rigaku MiniFlex600 benchtop X-ray diffraction (XRD) instrument (Rigaku Holdings Corporation, Tokyo, Japan) equipped with a position-sensitive D/teX Ultra detector and a cobalt tube. Diffraction patterns were recorded in the 2θ range of 10–90°, with a step size of 0.02° and a scan speed of 3°/min. The registered patterns were analysed using SmartLab Studio II software (Rigaku, Japan), with phase identification carried out using the PDF 2 release 2019 database (International Centre for Diffraction Data, Newtown Square, PA, USA). The particle size distribution of the powders was measured using a MasterSizer 3000 laser diffraction particle size analyser (Malvern Panalytical Ltd., Malvern, UK).

### 2.4. Differential Thermal Analysis

Differential thermal analysis (DTA) was conducted using the Setaram SETSYS 18 TM (TG/DTA/DSC) laboratory system equipped with a DTA “S” type measuring rod (thermocouple Pt/PtRh 10%). Temperature calibration was performed using high-purity silver (Ag, 99.999%) prior to the analysis. The samples were placed in 100 µL corundum crucibles, with an empty corundum crucible serving as the reference. The space surrounding the samples was flushed and evacuated three times before analysis. During the experiments, a dynamic argon atmosphere (99.9999%) was maintained within the furnace. Two experimental setups were employed:

- Cycling experiment: The sample was subjected to two heating-cooling cycles. In each cycle, the sample was heated from RT (20 °C) to 1310 °C at a rate of 10 °C/min and subsequently cooled back to RT at the same rate.
- Isothermal annealing: The sample was heated to 700 °C at a rate of 10 °C/min, held isothermally at this temperature for 8 h, and then cooled back to RT at the same rate.

### 2.5. Magnetic Measurements

The magnetic properties of prepared powders were obtained using a Physical Property Measurement System (PPMS, Dynacool, Quantum Design). The magnetization curves with maximal applied magnetic field ±4000 kA/m (±5 T) were measured at chosen constant temperatures between 5 K and 300 K. The zero field-cooled (ZFC) and field-cooled (FC) curves were acquired in the same temperature range in constant magnetic fields of 80 kA/m and 800 kA/m.

## 3. Results and Discussion

### 3.1. As-Milled Powder

**Figure 2:**
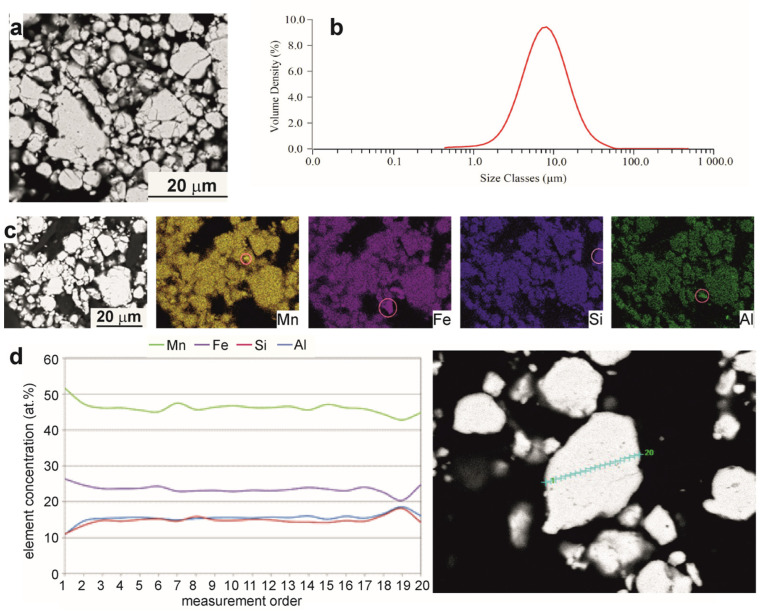
Microstructure of the as-milled Mn_2_FeSi_0.5_Al_0.5_ alloy powder. (**a**) SEM/BSE micrograph. (**b**) Particle size distribution. (**c**) X-ray maps of Mn, Fe, Si, and Al elements; localized regions with unincorporated alloying elements are marked with circles. (**d**) Line analysis through the volume of the chosen particle. Blue signs indicate the locations of the analysis.

The microstructure of the as-milled Mn_2_FeSi_0.5_Al_0.5_ alloy powder is shown in Figure 2. Figure 2a presents a SEM micrograph, confirming that the as-milled sample consists of numerous particles with an average size ranging between 5 and 15 µm.

The particles exhibit multiple cracks and chips, characteristic of the milling process. The particle size distribution, shown in Figure 2b, indicates a median diameter (D50) of approximately 7.7 µm. The homogeneous distribution of alloying elements throughout the particle volume after 168 h of milling was confirmed by X-ray elemental mapping (Figure 2c) and line analysis (Figure 2d). However, localized regions with unincorporated alloying elements were still observed (highlighted by red circles in the elemental maps of Mn, Fe, Si, and Al). Additionally, a slight deviation in the average chemical composition compared to the nominal composition was detected, as detailed in Table 1.

**Figure 3:**
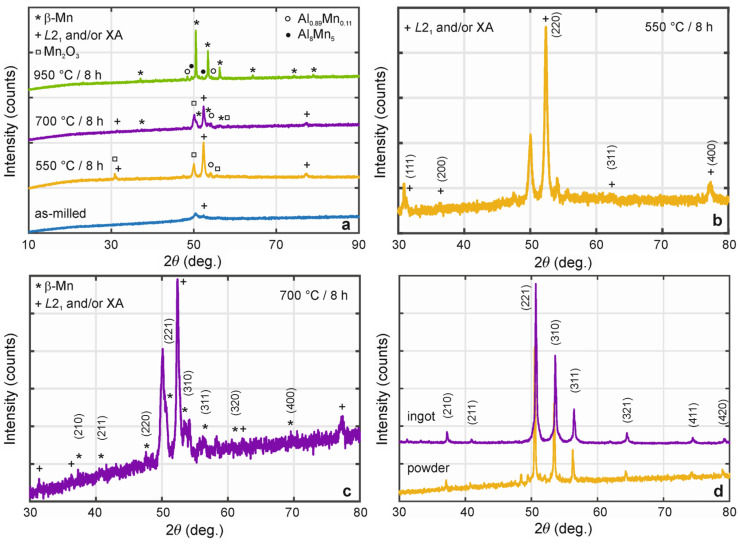
(**a**) X-ray diffraction patterns of as-milled and annealed Mn_2_FeSi_0.5_Al_0.5_ alloy powders. Separate diffractograms of samples annealed at 550 °C/8 h (**b**) and 700 °C/8 h (**c**) with marked Heusler and β-Mn reflections. (**d**) Comparison of diffractograms of Mn_2_FeSi_0.5_Al_0.5_ samples prepared by different technologies—alloy powder annealed at 950 °C/8 h and bulk ingot homogenized at 500 °C/100 h.

X-ray diffraction pattern in Figure 3a shows two not quite distinct peaks at angles 2θ ≈ 50° and 52°, the first one coming most probably from the n-hexane used and the second one identified as a cubic full-Heusler *L*2_1_ (Fm-3m) or inverse-Heusler XA (F-43m) structure. Similarly, as in the case of an Mn_2_FeSi alloy [20], additional annealing of the as-milled powder should contribute to diminishing of the n-hexane peak and to the homogenization and enhancement of the crystalline structure(s). The optimization of the annealing process was determined based on the results of the differential thermal analysis.

### 3.2. Differential Thermal Analysis

**Figure 4:**
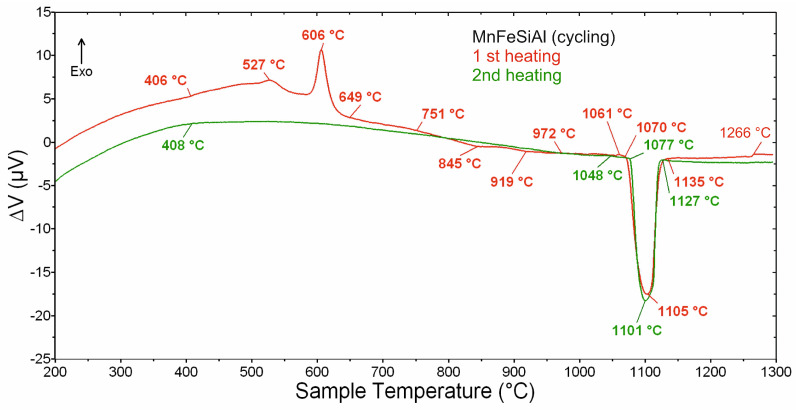
The results of DTA–cycling experiment.

Figure 4 presents the DTA results from the cycling experiment. During the first heating cycle (red curve), several exothermic and endothermic thermal effects were observed at both lower and higher temperatures. The first two exothermic effects, occurring in the temperature range of 406–510 °C, are relatively small and associated with a gradual release of heat. In contrast, the subsequent two exothermic effects are more pronounced, with peak temperatures at 527 °C and 606 °C, respectively. At higher temperatures, two small overlapping endothermic effects are observed within the range of 751–972 °C. The most significant endothermic effect, corresponding to the melting of the sample, occurs between 1061–1135 °C. Additionally, a “step-like” disturbance is detected at 1266 °C, likely caused by the movement of the molten sample within the crucible during measurement. The green curve, representing the second heating cycle, differs significantly. It displays only one prominent endothermic effect in the temperature range of 1048–1127 °C, which is also associated with the melting of the sample.

**Figure 5:**
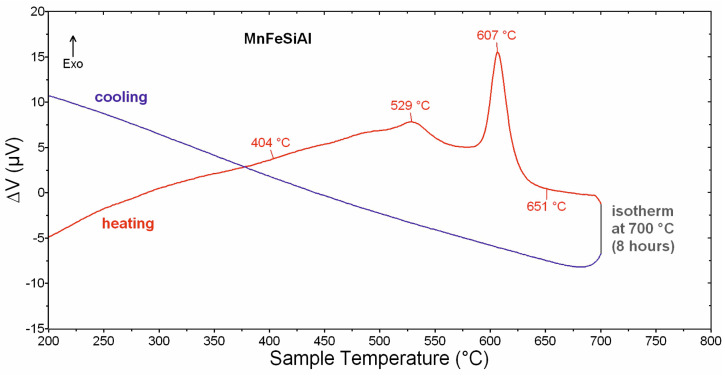
The results of DTA–isothermal annealing at a temperature of 700 °C for 8 h.

Figure 5 shows the DTA results for the isothermal annealing experiment conducted at 700 °C for 8 h. The DTA heating curve reveals four overlapping exothermic effects in the temperature range of 404–651 °C. These effects closely resemble those observed during the first heating cycle of the cycling experiment. However, no thermal effects are observed on the DTA cooling curve after the eight-hour annealing period at 700 °C.

The DTA results suggest that the formation of the final crystal structure in the Mn_2_FeSi_0.5_Al_0.5_ alloy powder begins at around 600 °C. This also implies that the Heusler structure, already present in the as-milled state, undergoes further modification during annealing. To validate this hypothesis, as-milled samples were annealed at different temperatures below and above 600 °C, with a detailed analysis of their structural and magnetic properties. The selected annealing temperatures were 550 °C, 700 °C, and 950 °C, as discussed in subsequent sections.

### 3.3. Annealed Powders

The measured XRD patterns of the annealed samples, shown in Figure 3a, differ significantly from those of the as-milled powder. Upon annealing at 550 °C for 8 h, the disappearance of the first peak (associated with n-hexane) and a notable strengthening of the second peak at 52.36°—corresponding to the Heusler structure—are observed. All five peaks related to the Heusler structure are marked with (+), see Figure 3b, and the calculated lattice constant is 0.5739 nm. This lattice constant is slightly higher than those reported for Mn_2_FeSi Heusler alloys prepared by other methods: 0.5677 nm for powders [20] and 0.5672 nm for ingots [22]. The observed shift is attributed to the presence of Al atoms in the quaternary alloy powder, whose atomic radius (0.125 nm) differs from that of Si atoms (0.110 nm).

In general, preparing alloys with a single-phase structure using powder metallurgy techniques is challenging. Heusler alloys are particularly sensitive to oxidation during mechanical alloying and subsequent annealing, especially at elevated temperatures. Oxygen, being highly reactive, readily interacts with the surface of alloy particles, leading to the formation of an oxide layer. This oxide layer can significantly affect the surface composition and morphology of the alloys, ultimately influencing their physical properties. To minimize oxidation, annealing is typically performed under an inert atmosphere, such as argon. However, despite employing a protective argon atmosphere, a manganese oxide phase was detected in the sample annealed at 550 °C for 8 h (Figure 3a). A notable peak at 50.12° (denoted by □) corresponds to the Mn_2_O_3_ cubic structure, with a lattice constant of 0.9464 nm. This observation suggests that the oxide phases may originate from the surface oxidation of the starting powders. Additionally, a minor peak (denoted by ○) was identified as a non-oxide tetragonal Al_0.89_Mn_0.11_ phase.

Consistent with these observations, more pronounced structural changes are evident upon increasing the annealing temperature to 700 °C, see detail in Figure 3c. New peaks (marked with *) appear, corresponding to the primitive cubic β-Mn structure with a lattice constant of 0.6281 nm. Similar to earlier findings, the lattice constant of the β-Mn structure in the quaternary alloy is slightly lower than that reported for Mn_2_FeAl alloys in the literature (~0.6339 nm [22]).

The transition from the Heusler to the β-Mn structure is complete after annealing at 950 °C. The peaks corresponding to the Heusler and Mn_2_O_3_ structures disappear entirely, and the β-Mn structure becomes dominant. These results align with those reported in ref. [25], where the microstructural and magnetic properties of quaternary Mn_2_FeSi_0.5_Al_0.5_ ingots prepared using induction melting were studied. Comparing the diffractograms of the powder and bulk samples (Figure 3d) reveals structural similarities, although the peaks for the ingot are slightly shifted, resulting in a slightly lower lattice constant (0.6274 nm). Nevertheless, the powder does not exhibit a single-phase structure due to the formation of small additional peaks identified as Al_8_Mn_5_ (rhombohedral structure) and Al_0.89_Mn_0.11_ (denoted by full and empty circles, respectively). Thus, the presence of minor parasitic structures persists throughout the entire annealing process, further complicating the complete stabilization of the alloy’s structure.

The observed impurity phases in the XRD patterns align well with deviations in Si and Al concentrations identified in the EDX analysis. Specifically, the reduction in Si content at 700 °C suggests phase segregation, likely resulting in the formation of Mn-rich and Al-rich phases, as evidenced in the diffraction patterns. These findings indicate incomplete homogenization during the annealing process, where the redistribution of alloying elements remains partial. The correlation between XRD and EDX analyses emphasizes the complex interplay between chemical composition, thermal treatment, and the stabilization of secondary phases. This underscores the necessity for further optimization of annealing conditions to achieve uniform element distribution and suppress impurity phases, ultimately enhancing the crystalline structure’s homogeneity.

**Figure 6:**
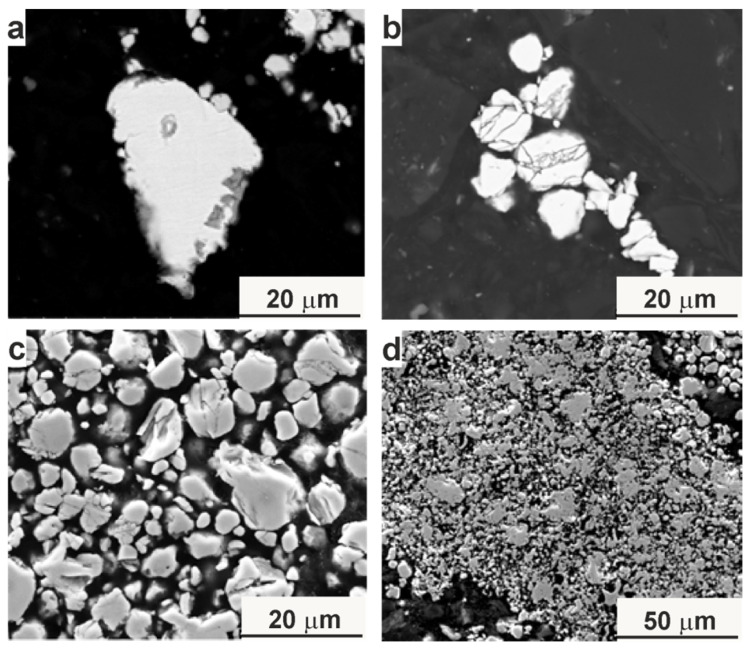
Microstructure of the annealed Mn_2_FeSi_0.5_Al_0.5_ alloy powders: (**a**) 550 °C/8 h, (**b**) 700 °C/8 h, (**c**) 950 °C/8 h, (**d**) individual particles and agglomerates sintered during annealing.

Figure 6 presents SEM micrographs of the annealed powders, which consist of particles with sizes similar to those of the as-milled sample (5–15 µm). The annealing process did not result in any melting of the powder mixture, as evidenced by the preservation of cracks and other defects. This is further confirmed by Figure 6c, where individual particles and agglomerates remain clearly distinguishable.

**Figure 7:**
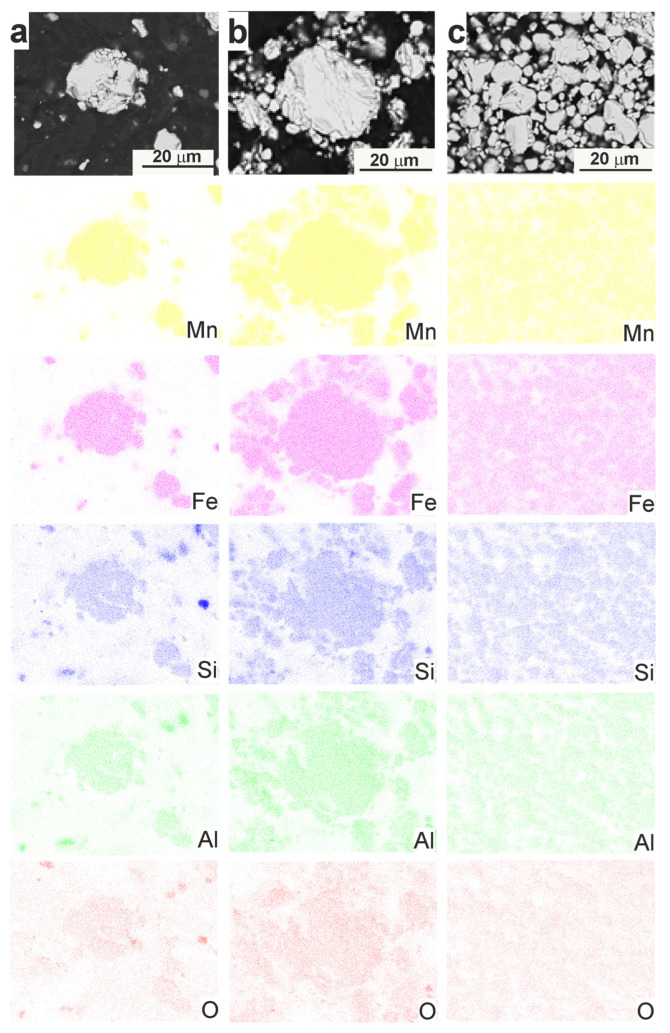
X-ray maps showing the distribution of Mn, Fe, Si, Al and O in the particle of Mn_2_FeSi_0.5_Al_0.5_ alloy powder after annealing at (**a**) 550 °C/8 h, (**b**) 700 °C/8 h, and (**c**) 950 °C/8 h.

Prolonged annealing facilitated the diffusion of all elements, leading to the formation of the β-Mn structure, as confirmed by XRD analysis. The development of this structure is a result of the mechanical alloying process, followed by partial synthesis during annealing. The distribution of alloying elements and oxygen within the particles is illustrated in Figure 7, highlighting the homogeneity achieved through the annealing process.

It is important to note that some of the detected oxygen may have originated from the metallographic preparation of the samples. Localized oxidation of silicon, likely resulting in the formation of silicon oxide (presumably SiO_2_), was observed in the powder alloy annealed at 550 °C (Figure 7a). This oxidation was confined to specific regions rather than being uniformly distributed throughout the alloy. Additionally, XRD analysis identified the presence of Mn_2_O_3_ (Figure 3a), indicating that a portion of the manganese had oxidized to form this oxide phase. However, the oxidation of manganese was not distinctly observable in the X-ray elemental maps, suggesting that it may be limited to specific microstructural features.

In the powder annealed at 700 °C, the Mn_2_O_3_ content was slightly lower compared to that in the powder annealed at 550 °C. This reduction is likely due to the formation of the β-Mn structure, where manganese previously present as Mn_2_O_3_ may have been incorporated into the β-Mn phase. This incorporation reduced the amount of free manganese available to form oxides. Silicon oxides were still observed (Figure 7b), but they appeared less prominently, possibly due to reduced oxidation or changes in the alloy’s composition and phase stability at this temperature.

After annealing at 950 °C, Mn_2_O_3_ was no longer detectable in the powder alloy (Figure 3a). This absence suggests that manganese was fully incorporated into the β-Mn structure or other newly formed phases, or that the oxidation conditions had changed, preventing the formation of manganese oxides. The appearance of intermetallic phases such as Al_0.9_Mn_0.1_ and Al_8_Mn_5_ indicates significant phase transformations. These intermetallic compounds likely resulted from the diffusion of aluminium and manganese at elevated temperatures, leading to the formation of stable intermetallic structures. This transformation suggests a shift from a solid solution or manganese oxide formation to more thermodynamically stable intermetallic phases.

**Figure 8:**
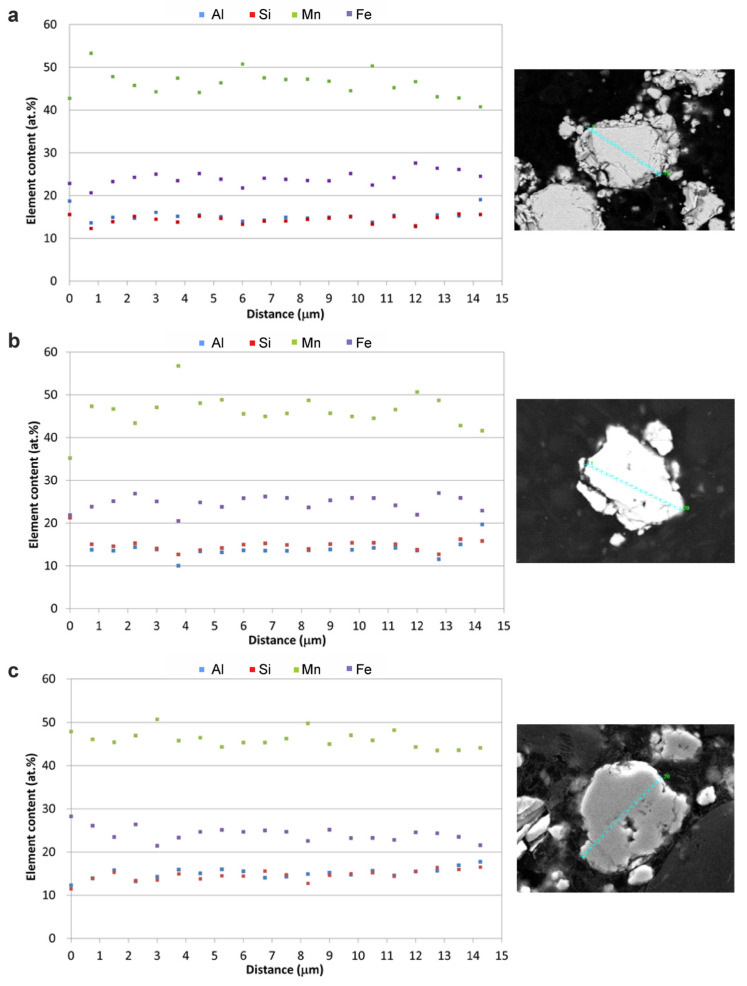
Line analysis through the volume of particles of Mn_2_FeSi_0.5_Al_0.5_ alloy powder after annealing at: (**a**) 550 °C, (**b**) 700 °C, and (**c**) 950 °C. Blue signs indicate the locations of the analysis.

Compared to the as-milled sample, the concentration profiles of annealed powders exhibited a sinusoidal pattern, particularly for Mn and Fe, as shown in Figure 8. The standard deviation of the line analysis revealed a significant reduction in Mn concentration dispersion, decreasing from ±3.4 at.% after annealing at 550 °C to ±1.9 at.% after annealing at 950 °C. Mn diffusion was notably more pronounced than that of the other alloying elements, especially during annealing at 700 °C. No unincorporated alloying elements were detected in the particle structure after annealing, except for the sample annealed at 550 °C, where residual silicon was observed. This residual silicon is likely due to its relatively high melting point, which may hinder complete reaction with other alloying elements within the given annealing duration. At lower annealing temperatures, kinetic limitations may prevent the full incorporation of silicon into the Heusler or β-Mn structure. The results of spot chemical analysis, summarized in Table 1, indicate that the average chemical composition of the powder annealed at 950 °C closely matches the nominal composition of the alloy.

**Figure 9:**
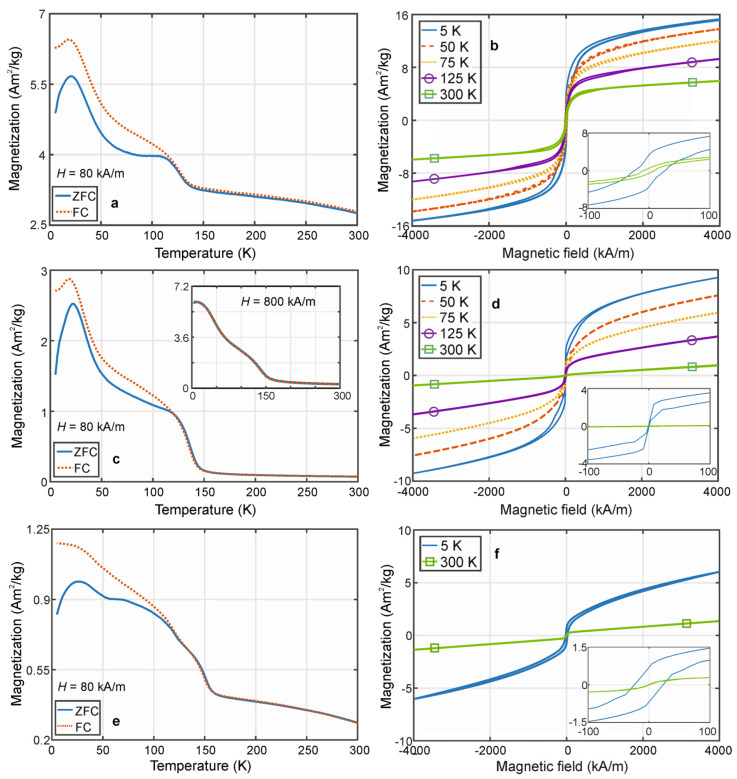
Low temperature magnetic properties of Mn_2_FeSi_0.5_Al_0.5_ alloy powders annealed at 550 °C/8 h (top line), 700 °C/8 h (middle line), and 950 °C/8 h (bottom line). Zero field-cooled (ZFC) and field-cooled (FC) curves measured in a constant magnetic field of 80 kA/m are presented in subplots (**a**,**c**,**e**). Magnetization curves at chosen constant temperatures are shown in subplots (**b**,**d**,**f**). Details of magnetization curves (5 K, 300 K) at low magnetic fields are presented in insets.

Magnetic properties of annealed alloy powders are presented in Figure 9. The magnetic behaviour of the powders annealed at 550 °C/8 h is shown in Figure 9a,b. The room temperature magnetization curve (300 K) consists of magnetization reversal at lower magnetic fields with nonzero remanence and coercive field and a non-saturated linear part at higher magnetic fields indicating magnetization disorder. Similar magnetization curves were presented for Mn_2_FeSi alloy powder with the Heusler structure [20]; however, the magnetization reversal was significantly weaker. This difference could be attributed to the influence of Al atoms or the presence of impurities and parasitic structures, such as manganese oxides, in the quaternary alloy. For the single Heusler structure (Mn_2_FeSi), the paramagnetic-antiferromagnetic behaviour is typically observed at low temperatures. However, this pure structure is obtained for bulk samples—ingots—prepared by arc or induction melting techniques. The ZFC/FC curves of quaternary Mn_2_FeSi_0.5_Al_0.5_ alloy powder measured during cooling from room temperature to 20 K under an applied field of H = 80 kA/m (Figure 9a) reveal multiple transitions to ferro-/ferrimagnetic and paramagnetic states, with a separation of the curves around 120 K. These transitions reflect the existence of several magnetic phases (impurities), which are also evident in the low-temperature magnetization curves (Figure 9b). The decrease in magnetization on the ZFC/FC curves below 20 K indicates possible antiferromagnetic behaviour. For sake of comparison, the antiferromagnetic properties of bulk and powder Mn_2_FeSi Heusler systems were observed below 50 K [21,22] and 67 K [20], respectively. This suggests that Al atoms influence the shift in the peak position of the ZFC/FC curves towards lower temperatures. Additionally, the low-temperature magnetization curves exhibit an increase in remanent magnetization, coercive field, and magnetization at higher magnetic fields as the temperature decreases.

Because of the still dominant Heusler structure, the alloy powder annealed at 700 °C/8 h (Figure 9c,d) does not experience significant changes in magnetic properties compared to the previous sample. Decrease in magnetization is observed below 23 K, with the separation of the ZFC/FC curves occurring around 120 K. However, the emergence of the highly frustrated cubic β-Mn structure gradually impacts two key aspects of the system’s magnetic properties. First, the system is fully paramagnetic at room temperature (RT) and remains in this state during cooling until approximately 135 K. The transition to an ordered phase occurs below this temperature, as indicated by the magnetization curves. As the temperature decreases to 5 K, the remanent magnetization and coercive field progressively increase reflecting probably the influence of impurities. Second, the magnetization values are overall lower than those of the previous sample. Specifically, the magnetization values, determined from the magnetization curves at a maximum magnetic field of 4000 kA/m, are 1.5 to 5 times lower at different temperatures. This highlights the high sensitivity of the magnetic properties of these systems to minor structural changes. The inset of Figure 9c shows the ZFC/FC curves measured at a higher magnetic field of 800 kA/m. While the shape of the FC curve remains nearly unchanged, the ZFC curve closely follows the FC curve without any splitting. The peak of the ZFC/FC curves is significantly shifted to lower temperatures (≈8 K), which points to the possibility of controlling the low-temperature magnetic properties of the alloy powder through an external magnetic field.

The results of XRD measurements confirmed the transition from the Heusler to the β-Mn structure after annealing for 8 h at a temperature of 950 °C. This structural transformation significantly impacts the magnetic characteristics, as shown in Figure 9e,f. The linear dependence of magnetization on the applied external magnetic field observed in the room-temperature (300 K) magnetization curve confirms the paramagnetic nature of the powder alloy. At the same time, the reversal of magnetization at small magnetic fields is also evident on this loop, indicating the existence of a ferro-/ferrimagnetic contribution at RT. Similar to the powder annealed at 550 °C for 8 h, the ZFC/FC curves measured at 80 kA/m during cooling below 300 K deviate from the Curie-Weiss law, with the ferro-/ferrimagnetic phase dominating. A jump in magnetization occurs at a temperature just above 150 K. Between 150 K and 25 K, ferro-/ferrimagnetic behaviour continues to prevail, accompanied by several magnetic transitions and splitting of the ZFC/FC curves, likely caused by minor structural changes in the β-Mn structure. The antiferromagnetic behaviour observed below 25 K agrees well with the previous samples. On the contrary, the magnetization values decreased in comparison to previous samples reflecting the highly frustrated nature of β-Mn structure. Note that the described low-temperature magnetic properties of alloy powder are different from the ingot of the same composition having dominant β-Mn structure [25]. The ingot exhibits typical paramagnetic-antiferromagnetic behaviour with a Néel temperature 34–37 K. Given the low content of parasitic structures (~6 wt.%), it appears that the atomic arrangement within the β-Mn structure is highly sensitive to the preparation method, which fundamentally influences the magnetic properties of these materials.

## 4. Conclusions

In this paper, we present the structural and magnetic properties of the quaternary Mn_2_FeSi_0.5_Al_0.5_ alloy powder, successfully synthesized from pure elements via mechanical alloying. The as-milled powder (168 h of milling) consisted of particles with an average size 5–15 µm, though its structure was characterized by only two small, sharp peaks in the XRD pattern. The annealing temperatures of 550 °C/8 h, 700 °C/8 h, and 950 °C/8 h were chosen based on the results of DTA analysis. The homogeneous distribution of elements and similar particle morphologies were observed across all annealing temperatures. However, the structural changes documented in the XRD spectra revealed the following: (i) a gradual transformation from the cubic Heusler structure to the primitive cubic β-Mn structure with increasing annealing temperature, (ii) a higher concentration of manganese oxides at lower annealing temperatures (550 °C and 700 °C), and (iii) significant effects on the low-temperature magnetic properties of all prepared samples. The ZFC/FC curves measured under a magnetic field of 80 kA/m demonstrated antiferromagnetic behaviour below 20 K for all samples, with a separation of the curves observed at 120–130 K. For samples with a dominant Heusler structure (annealed at 550 °C and 700 °C), paramagnetic behaviour was predominant in the temperature range of 20–120 K, although several ferro-/ferrimagnetic transitions were also detected. The room temperature magnetization curve of alloy powder annealed at 950 °C having a β-Mn structure showed paramagnetic contribution at higher magnetic fields and a magnetization reversal at lower magnetic fields. The presence of the ordered phase seemed to be dominant during cooling from RT to 25 K, where the peak of the ZFC/FC curves was identified. This sample exhibited the lowest magnetization values at different temperatures and magnetic fields, confirming the high frustration of β-Mn structure.

## Figures and Tables

**Table 1:** The averaged chemical analysis of Mn_2_FeSi_0.5_Al_0.5_ alloy powder in as-milled state and after annealing at 550 °C, 700 °C, and 950 °C (averaged values from seven-spot EDX analysis). The values after the plus/minus sign represent the standard deviations of the seven-point measurements.

Sample	Mn	Fe	Si	Al
	(at.%)			
nominal composition	50.0	25.0	12.5	12.5
as-milled state	46.1 ± 0.5	24.1 ± 0.4	14.6 ± 0.3	15.2 ± 0.3
annealed at 550 °C/8 h	45.5 ± 1.4	24.0 ± 0.3	15.4 ± 1.3	15.2 ± 0.5
annealed at 700 °C/8 h	51.8 ± 1.9	24.3 ± 3.3	10.2 ± 1.5	13.7 ± 2.2
annealed at 950 °C/8 h	49.0 ± 1.8	24.3 ± 0.6	13.0 ± 0.9	13.7 ± 1.0

## Data Availability

The data that support the findings of this study are available from the corresponding author (Katerina Skotnicova) upon request due to privacy.
